# Predictive modeling of axillary web syndrome in Chinese postoperative breast cancer patients using interpretable machine learning

**DOI:** 10.3389/fonc.2026.1900340

**Published:** 2026-09-03

**Authors:** Jiali Du, Jing Yang, Xujin Chen, Zhiyan Cheng, Tong Xiang

**Affiliations:** Department of Breast Surgery, Sichuan Clinical Research Center for Cancer, Sichuan Cancer Hospital &Institute, Sichuan Cancer Center, Affiliated Cancer Hospital of University of Electronic Science and Technology of China, Chengdu, China

**Keywords:** axillary web syndrome, breast cancer, machine learning, prediction model, risk factors

## Abstract

**Objective:**

This study aimed to develop and validate a series of risk prediction models for axillary web syndrome (AWS) after breast cancer surgery using machine learning algorithms, enabling postoperative risk identification to facilitate timely targeted interventions.

**Methods:**

This retrospective cross-sectional study was conducted at a tertiary cancer hospital between April 2021 and July 2024. After collecting clinical data, the least absolute shrinkage and selection operator (LASSO) regression was employed to screen variables with predictive value for AWS. The dataset was randomly divided into a development set and a test set at a 7:3 ratio. Nine classification models were constructed, and their performance was evaluated based on accuracy, precision, recall, F1-score, and area under the curve (AUC). The optimal model was selected according to the AUC value. Model interpretation was performed using SHAP analysis and feature importance ranking.

**Results:**

A total of 655 patients were included, with 459 in the development set and 196 in the test set. The AWS incidence rate was 26.9% (176 cases). LASSO regression identified 14 most predictive features, and SHAP value analysis revealed that lymph node surgical approach and lymphedema were the most critical variables. The logistic regression (LR) model demonstrated the best overall predictive performance (AUC = 0.72).

**Conclusion:**

This study confirmed that the LR model exhibited superior accuracy and AUC value combinations, effectively identifying high-risk AWS patients. The findings provide a basis for clinical practitioners to identify high-risk patients and implement targeted interventions.

## Introduction

According to the latest GLOBOCAN 2020 data, breast cancer has surpassed lung cancer as the most common malignancy in women, with approximately 2.3 million new cases annually worldwide, accounting for 11.7% of all cancer cases (1). Significant advancements in breast cancer treatment have improved both overall and relative survival rates. Currently, the 5-year survival rate for breast cancer reaches 90%, significantly higher than that of other malignancies, indicating its gradual transition to a chronic disease (2).

With the improvement in breast cancer survival rates, enhancing patients’ quality of life has become increasingly important. Axillary web syndrome (AWS) is a common yet frequently overlooked complication, typically occurring subsequent to axillary lymph node surgery for breast cancer (e.g., complete axillary lymph node dissection or sentinel lymph node biopsy) (3). This syndrome is characterized by palpable or visible cord-like subcutaneous structures, predominantly forming in the axillary region and potentially extending along the inner arm to the antecubital fossa and wrist (4). The pathophysiological mechanisms underlying AWS remain incompletely understood, though it is widely hypothesized that lymphatic system damage caused by axillary surgery may be the primary trigger. Specifically, surgical intervention disrupts normal axillary lymphatic drainage pathways, leading to abnormal lymph fluid reflux, subsequent perivascular tissue fibrosis, and ultimately the formation of cord-like structures (5). AWS may cause discomfort in the affected limb and restrict range of motion, potentially resulting in long-term complications such as myofascial pain syndrome, muscle atrophy, and periarthritis of the shoulder, significantly impairing patients’ quality of life and long-term prognosis (6, 7). However, reported occurrence rates of AWS vary widely (6%–91%) (8). Although the incidence of AWS has shown a declining trend in recent years, its management remains a significant clinical challenge. The current lack of effective AWS treatment strategies underscores the need for preventive measures to reduce its occurrence.

Machine Learning(ML) as a service enables clients to leverage the advantages of machine learning by providing developers with rapid and efficient access to pre-designed algorithms and models, eliminating the time, financial investment, and potential risks associated with establishing an in-house machine learning team (9). In recent years, the application of machine learning in medicine has grown significantly (10), with its use extending to the development of precise predictive models. Compared to traditional statistical techniques, machine learning more effectively identifies variables associated with clinical outcomes, offers superior predictive performance, more accurately simulates complex relationships, and learns from diverse data modules (11). Although numerous studies have focused on constructing AWS predictive models, most still rely on conventional methods such as logistic regression. The field of machine learning encompasses various algorithm categories, including K-Nearest Neighbor (KNN), Stochastic Gradient Descent (SGD), and Gradient Boosting Machines (GBM). Given the inherent differences in the predictive efficacy of models built using different algorithms, a comparative study of AWS predictive models is warranted.

This study aims to develop and validate a series of predictive models specifically designed to assess AWS risk in the postoperative setting, utilizing large-scale retrospective cross-sectional data for validation. These models may assist healthcare professionals in identifying patients at higher risk for AWS postoperatively, facilitating timely targeted interventions.

## Materials and methods

This retrospective cross-sectional study enrolled consecutive breast cancer cases diagnosed between 2021 and 2024. The study protocol was approved by the Ethics Committee of Sichuan Cancer Hospital (Approval No.: SCCHEC-02-2024-247). All procedures were conducted in accordance with the Declaration of Helsinki and relevant ethical guidelines. Informed consent was obtained from all participants prior to their inclusion in the study.

### Study design

A retrospective cross-sectional study was conducted at Sichuan Cancer Hospital, the largest oncology diagnosis and treatment center in Southwest China, to ensure sufficient sample size. The data were accessed for research purposes from 1 April 2021 to 1 July 2024. Inclusion criteria were as follows: (1) age ≥18 years at the time of surgery; (2) histopathologically confirmed breast cancer with surgical treatment; (3) normal reading, comprehension, and communication abilities; and (4) voluntary participation in the study. Exclusion criteria included: (1) preexisting shoulder conditions causing pain and restricted mobility; (2) history of axillary or shoulder surgery on the affected side; and (3) bilateral breast cancer.

### Data collection process

Patient demographics, clinical data, and treatment information (**Table 1**) were retrieved from the electronic medical record system. To ensure comprehensiveness and accuracy, two researchers independently collected and cross-verified the data, with discrepancies resolved by a third researcher for final confirmation. All variables and AWS status were assessed within 3 months after surgery. Adjuvant chemotherapy and radiotherapy were initiated after surgery and may have occurred after AWS onset. These variables were included in the model as exploratory clinical factors.

**Table 1:** Categories and variables from the study applied in predicting AWS.

Category	Variables
Demographic characteristics	age, Body Mass Index(BMI),hypertension, diabetes
Clinical data	tumor site, clinical staging
Treatment types	type of surgery, type of lymph node surgery, number of removed lymph nodes, number of positive lymph nodes, neoadjuvant chemotherapy, adjuvant chemotherapy, radiotherapy
Postoperative Complications	lymphoedema, shoulder disorder

### Evaluation of outcome

Diagnostic Criteria for Axillary Web Syndrome (AWS):AWS diagnosis was established through physical examination by three certified lymphedema therapists using standardized protocols (7, 12), with unified training prior to the study. Patients were positioned supine with the affected upper limb maximally abducted. The presence of palpable cord-like nodules radiating from the axilla to the medial arm or chest wall, with or without localized skin discoloration, pain, or restricted mobility, was considered diagnostic of AWS. The exact onset date was recorded.

### Statistical analysis

AWS diagnosis was recorded based on physical examination findings at these scheduled visits. All AWS cases were identified within 3 months after surgery, consistent with the known temporal pattern of AWS onset. Complete follow-up data were available for all 655 patients, as clinical information was obtained from the electronic medical record system for patients who completed routine postoperative care at our institution. Missing data were minimal (<2% for any variable) and were handled using complete-case analysis. Data entry was performed using Microsoft Excel 2021, while statistical analysis was conducted using Python (version 3.12.3) and the Table One package (version 0.7.12). Continuous variables were expressed as mean ± standard deviation, and categorical variables were described using frequencies and percentages. Predictive models were constructed using Python (version 3.12.3) based on the following algorithms: logistic regression (LR), K-nearest neighbors (KNN), stochastic gradient descent (SGD), decision tree (DT), Random Forest (RF), support vector machine (SVM), adaptive boosting (AdaBoost), extreme gradient boosting (XGBoost), and gradient boosting machine (GBM). The dataset was randomly divided into a development set (70%) and a test set (30%). Model performance was evaluated using 5-fold cross-validation, with discriminative ability assessed via the area under the curve (AUC), accuracy, precision, recall, and F1-score. Hyperparameters for the LR model were set as follows: C = 1.0, penalty = ‘l2’, solver = ‘lbfgs’. Random seed was set to 42 for data splitting. To evaluate model calibration, we generated calibration plots comparing predicted probabilities against observed outcomes in the test set, complemented by the Hosmer-Lemeshow goodness-of-fit test. Decision curve analysis was performed to quantify the clinical net benefit of the logistic regression model across a range of threshold probabilities, comparing model-guided decisions against “treat-all” and “treat-none” strategies (13). These analyses were conducted using Python’s scikit-learn and scikit-survival libraries.

### Study results

#### Patient enrollment and baseline characteristics

Based on AWS assessment, 655 post-operative breast cancer patients were categorized into non-AWS (479 cases, 73.1%) and AWS (176 cases, 26.9%) groups. Demographic characteristics, clinical data, and treatment modalities are detailed in **Table 2**. Surgical factor comparisons revealed that the AWS group had a higher proportion of patients subjected to mastectomy (74.4% vs. 63.5%, p=0.031) and axillary lymph node dissection (ALND) (52.3% vs. 30.3%, p<0.001), along with a greater mean number of dissected lymph nodes (11.9 vs. 9.9, p=0.003). Postoperative complications were significantly more prevalent in the AWS group, including lymphedema (22.9% vs. 5.0%, p<0.001) and shoulder dysfunction (17.1% vs. 3.3%, p<0.001).

**Table 2:** Comparison of demographic characteristics, clinical data and, treatment types and postoperative complications of postoperative breast cancer patients between different groups.

Variable	Variable hierarchy	Total	Non-AWS	AWS	P value
		(n=655)	(n=479)	(n=176)	
Age, mean (SD)		51.16 (9.42)	51.48 (9.64)	50.3 (8.75)	0.1545
BMI,mean (SD)		23.94 (3.3)	24.11 (3.38)	23.47 (3.03)	0.027
Hypertension, n (%)	No	567 (86.56)	418 (87.27)	149(84.66)	0.4607
	Yes	88 (13.44)	61 (12.73)	27 (15.34)	
DM, n (%)	No	620 (94.66)	455 (94.99)	165 (93.75)	0.6677
	Yes	35 (5.34)	24 (5.01)	11 (6.25)	
tumor_site, n (%)	Left	338 (51.60)	241 (50.31)	97 (55.11)	0.3165
	Right	317 (48.40)	238 (49.69)	79 (44.89)	
clinical_staging, n (%)	0	3(0.46)	2(0.42)	1(0.57)	0.1435
	I	215(32.82)	168(35.07)	47(26.70)	
	II	357(54.50)	257(53.65)	100(56.82)	
	III	67(10.23)	42(8.77)	25(14.20)	
	IV	13(1.99)	10(2.09)	3(1.70)	
Type_of_surgery, n (%)	Breast-conserving surgery	215(32.83)	171 (35.70)	44 (25.00)	0.0311
	mastectomy	435 (66.41)	304 (63.47)	131 (74.43)	
	Breast reconstruction	5 (0.76)	4 (0.84)	1 (0.57)	
Type_of_lymph_node_surgery, n (%)	SLNB	418 (63.82)	334 (69.73	84 (47.73)	<0.001
	ALND	237 (36.18)	145 (30.27)	92 (52.27)	
Number_of_removed_lymph_nodes, mean (SD)		10.45 (7.4)	9.94 (7.39)	11.86 (7.15)	0.003
Number_of positive_lymph_nodes, mean (SD)		1.24 (3.02)	1.07 (2.87)	1.69 (3.36)	0.0194
Neoadjuvant_chemotherapy, n (%)	No	602 (91.91)	436 (91.02)	166 (94.32)	0.2266
	Yes	53 (8.09)	43(8.98)	10 (5.68)	
Adjuvant_chemotherapy, n (%)	No	219 (33.4)	15 (3.13)	4 (2.27)	0.7505
	Yes	436 (66.6)	464 (96.87)	172 (97.73)	
Radiotherapy, n (%)	No	333 (50.84)	242 (50.52)	91 (51.70)	0.857
	Yes	322 (49.16)	237 (49.48)	85(48.30)	
Lymphoedema, n (%)	No	589(89.92)	455(94.99)	134(76.14)	<0.001
	Yes	66(10.08)	24(5.01)	42(23.86)	
Shoulder_disorder, n (%)	No	609(92.98)	463(96.66)	146(82.95)	<0.001
	Yes	46(7.02)	16(3.34)	30(17.05)	

Feature selection was performed using LASSO regression, with optimal model parameters determined through cross-validation. A total of 14 features demonstrating the highest predictive value for axillary web syndrome (AWS) were identified.

**Figure 1:**
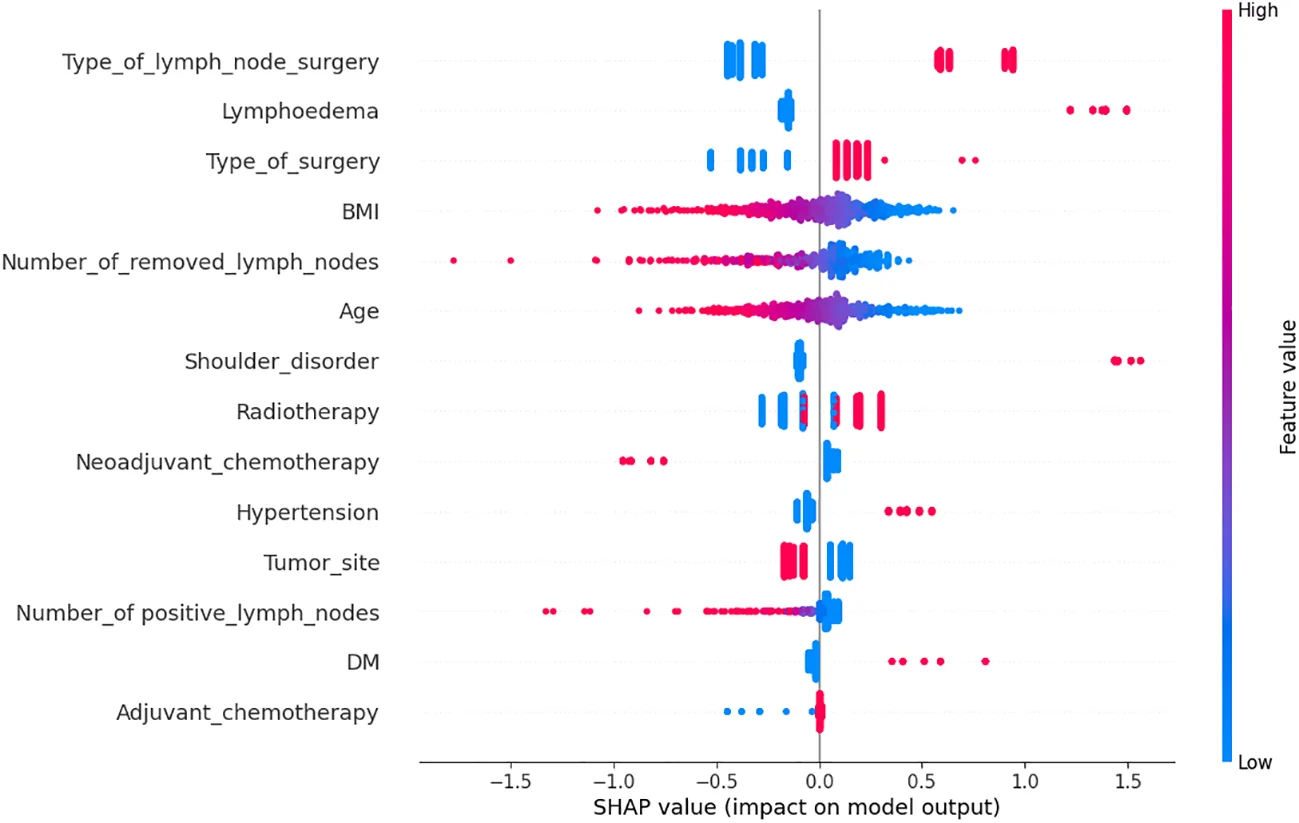
SHAP Value analysis for predictive modeling of AWS.

**Figure 1** illustrates the distribution of feature importance based on SHAP values, untangling the directional machine learning heterogeneity of variables in predicting postoperative AWS in breast cancer patients. Notably, type of lymph node surgery and lymphedema exhibited SHAP values predominantly concentrated in the positive region.

**Figure 2:**
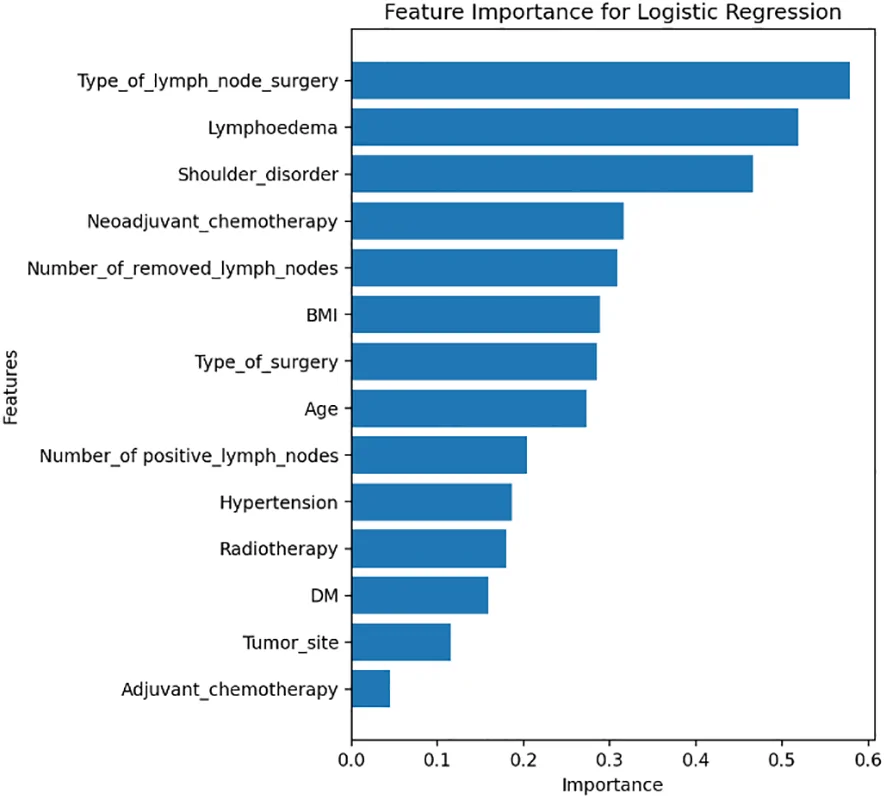
Importance ranking of variables.

**Figure 2** presents the ranking of feature importance derived from logistic regression analysis, reflecting the global contribution of each variable to AWS prediction. Type of lymph node surgery and lymphedema ranked first and second, respectively, in importance—a finding that aligns closely with their central role in SHAP analysis. These results collectively confirm the dominant influence of lymphatic system-related factors in the pathophysiology of AWS.

### Predictive performance of ML models

In the development set, modeling was performed using logistic regression (LR), k-nearest neighbors (KNN), stochastic gradient descent (SGD), decision tree (DT), Random Forest (RF), support vector machine (SVM), adaptive boosting (AdaBoost), extreme gradient boosting (XGBoost), and gradient boosting machine (GBM). Subsequently, internal validation was conducted using the test set. Model performance was evaluated through 5-fold cross-validation, with metrics including the area under the curve (AUC), accuracy, precision, recall, and F1-score to compare the predictive capabilities of different algorithms.

**Figure 3:**
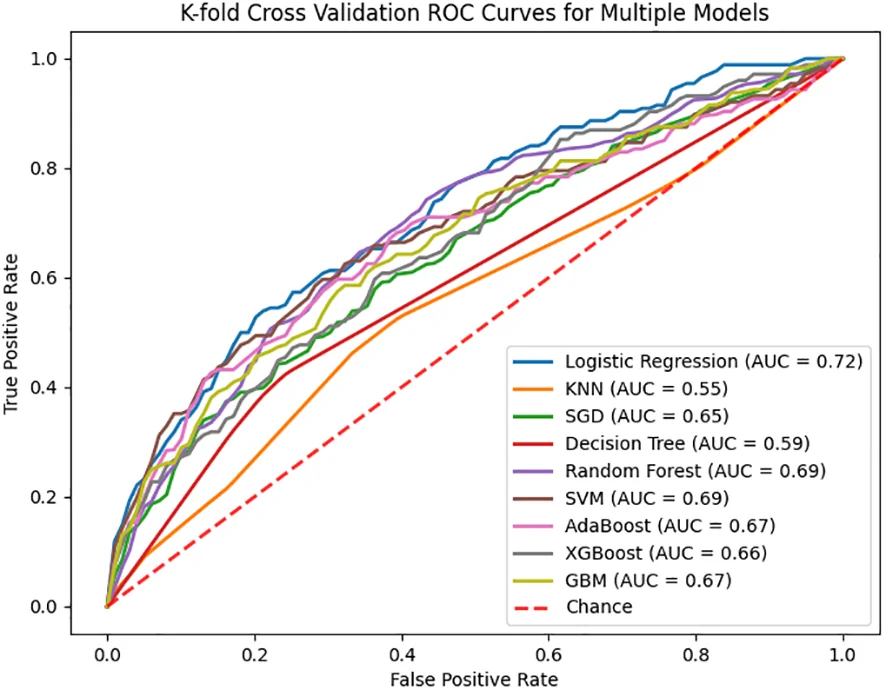
Receiver operating characteristic curve of the nine models in the test set.

Following comprehensive analysis, **Figure 3** presents the receiver operating characteristic (ROC) curves of the nine models. After internal validation for each model type, the performance metrics of all nine models were systematically compared. Based on the AUC value (0.72) and accuracy (76%), the logistic regression model was determined to exhibit the optimal predictive performance. **Table 3** summarizes the performance characteristics of these nine machine learning (ML) models in predicting axillary web syndrome (AWS) within the test set.

**Table 3:** Performance results for the machine learning models in the test set.

Model	Accuracy	Precision	Recall	F-score	AUC
LR	0.76	0.63	0.28	0.38	0.72
KNN	0.69	0.34	0.17	0.23	0.55
SGD	0.45	0.16	0.60	0.25	0.65
DT	0.67	0.39	0.39	0.39	0.59
RF	0.73	0.50	0.27	0.35	0.69
SVM	0.73	0.00	0.00	0.00	0.69
AdaBoost	0.75	0.58	0.29	0.38	0.67
XG Boost	0.71	0.43	0.33	0.37	0.66
GBM	0.74	0.52	0.30	0.37	0.67

We further evaluated the logistic regression model using additional performance metrics. The model achieved a PR-AUC of 0.53, balanced accuracy of 0.61, PPV of 0.63, and NPV of 0.78. The confusion matrix showed 49 true positives, 127 false negatives, 33 false positives, and 446 true negatives.

### Model calibration and clinical utility

**Figure 4:**
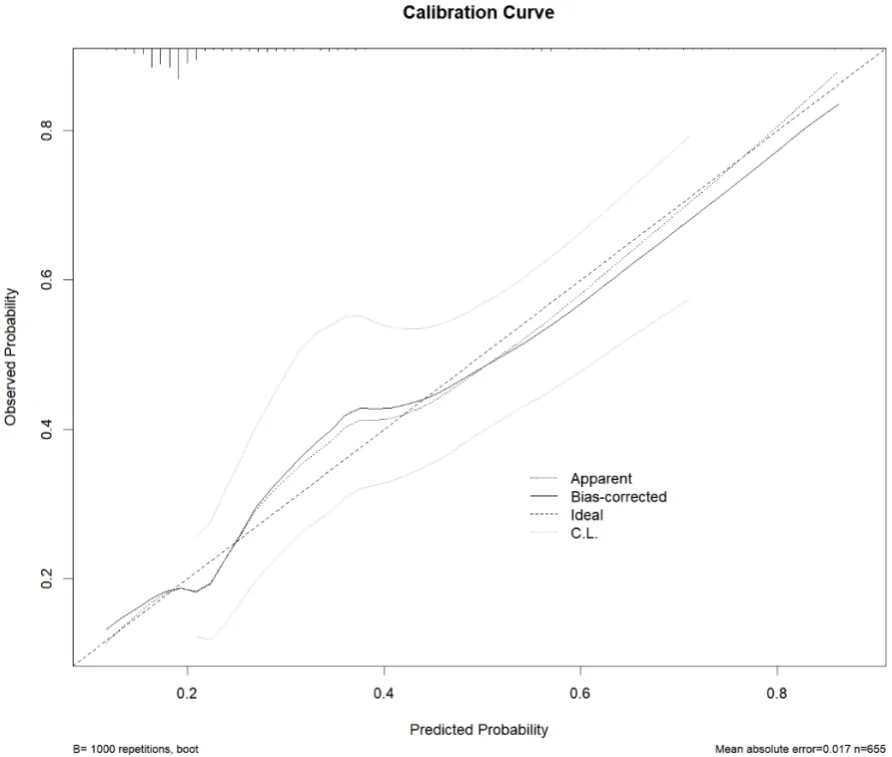
Calibration curve for the logistic regression model in the test set.

In addition to discrimination metrics, we evaluated the calibration and clinical utility of the logistic regression model. The calibration curve demonstrated good agreement between predicted probabilities and observed AWS outcomes (**Figure 4**), with a non-significant Hosmer-Lemeshow test (χ² = 8.24, p = 0.41), indicating adequate model fit.

**Figure 5:**
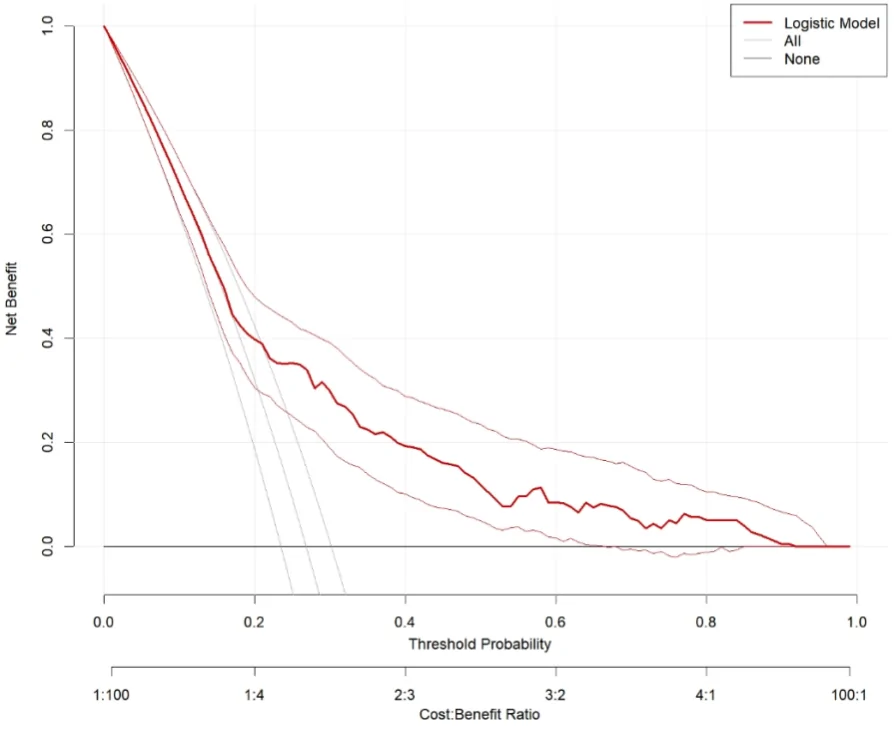
Decision curve analysis for the logistic regression model.

Decision curve analysis was performed to assess the clinical net benefit of the model across different threshold probabilities. As shown in **Figure 5**, the logistic regression model provided positive net benefit compared to both “treat-all” and “treat-none” strategies across a clinically relevant range of threshold probabilities (10% to 45%). These findings suggest that using the model to guide intervention decisions would yield better clinical outcomes than alternative strategies, supporting its potential utility in postoperative risk stratification.

## Discussion

This study demonstrated that 26.9% of breast cancer patients developed AWS postoperatively, a finding consistent with the incidence rates reported by some researchers (14–16). A retrospective analysis of 207 unilateral breast cancer patients subjected to mastectomy combined with lymph node dissection revealed an overall AWS incidence of 48.8%, with the rate reaching as high as 86% in those who underwent breast reconstruction (17). Another prospective study evaluating 116 patients who received axillary lymph node dissection (ALND) reported an AWS incidence of 48.3% (95% CI: 38.9–57.7) (18). The discrepancies among these studies may stem from variations in patient characteristics, surgical approaches, and follow-up durations, yet all indicate a substantial risk of AWS following breast cancer surgery. Consequently, identifying effective strategies to mitigate postoperative AWS risk has become a critical focus for clinicians. By elucidating the factors associated with AWS, its occurrence can be better understood, and targeted interventions can be implemented. In this study, LASSO regression was employed to screen 14 characteristic indicators.

This study identified 14 features through LASSO regression, among which the type of lymph node surgery and lymphedema demonstrated the highest contribution in SHAP analysis, consistent with the significant intergroup differences shown in **Table 2** (both p<0.001). However, factors such as hypertension, diabetes, and tumor site did not show significant intergroup differences in this study cohort (**Table 2**), resulting in lower predictive weights in the present model. These findings suggest that risk factors for AWS may exhibit heterogeneity across different populations or healthcare settings, warranting further validation in multicenter cohorts.

The type of lymph node surgery emerged as the most profound risk factor. Multiple studies have compared the incidence of AWS between patients undergoing sentinel lymph node biopsy (SLNB) and those subjected to ALND (19–21), consistently demonstrating a lower AWS rate in the SLNB group. A meta-analysis by Harris (22) confirmed that the risk of AWS after ALND was 1.7–7 times higher than that after SLNB. Compared with SLNB, ALND involves a more extensive surgical field, increasing the risk of damage to the breast, axillary lymphatic vessels, and superficial veins, which may lead to complications such as lymphatic vessel injury and superficial venous occlusion. These complications can impair lymphatic drainage, exacerbate systemic inflammatory responses, and predispose patients to superficial thrombophlebitis and lymphangitis, thereby elevating postoperative AWS risk (23, 24). Clinicians should strictly adhere to ALND indications, reduce its application in lymph node-negative patients, and minimize surgical trauma to the breast, axillary lymphatic vessels, and superficial veins to further mitigate AWS risk.

This study identified postoperative lymphedema as a significant risk factor for AWS in breast cancer patients. A strong association exists between AWS and post-breast cancer surgery lymphedema. Research indicates that lymphedema is one of the risk factors for AWS (25). A retrospective analysis of 354 breast cancer patients receiving physical therapy post-treatment showed that approximately one-third developed AWS, predominantly within 8 weeks post-surgery. Patients aged ≥60 years had a 73% higher risk of AWS than younger patients (OR = 1.73). Additionally, AWS patients exhibited a 44% increased risk of developing lymphedema within one year postoperatively (RR = 1.44). Notably, if AWS occurred within the first postoperative month, these patients had a nearly threefold higher risk of lymphedema within three months compared to other AWS patients (RR = 2.75) (26). Furthermore, numerous studies consistently confirm that ALND is a shared risk factor for both AWS and lymphedema. For instance, a retrospective analysis of 175 breast cancer patients who underwent ALND revealed a 20% incidence of lymphedema, with smoking, radiotherapy, and dissection of >18 axillary lymph nodes significantly elevating the risk (27). Similarly, AWS incidence was closely associated with ALND. A prospective cohort study of 193 breast cancer surgery patients demonstrated that ALND recipients were more prone to AWS (28). These findings underscore the need to recognize the link between postoperative AWS and lymphedema in breast cancer patients and to enhance monitoring and timely interventions for those with relevant risk factors.

There is a significant correlation between postoperative shoulder disorder and AWS. Patients with AWS frequently exhibit restricted shoulder mobility, primarily manifested as reduced range of motion (ROM), including limitations in shoulder abduction and forward flexion. A follow-up study of post-mastectomy patients demonstrated that during the early postoperative period (2–4 weeks), AWS patients showed significantly lower shoulder abduction ROM compared to non-AWS patients, with the difference being statistically significant (P < 0.05) (21). This may be attributed to the cord-like structures of AWS restricting normal movement of periarticular muscles and fasciae, thereby impairing functional mobility. Furthermore, shoulder dysfunction may exacerbate AWS symptoms, forming a vicious cycle. For instance, prolonged shoulder immobility can aggravate axillary tissue adhesion and fibrosis, making AWS cords more prominent and intensifying pain and mobility restrictions. Thus, early assessment and intervention for shoulder function are critical for AWS prevention and management in post-mastectomy patients.

This study suggests a potential association between neoadjuvant chemotherapy and AWS development. Multiple studies indicate that patients subjected to neoadjuvant chemotherapy may exhibit an elevated incidence of AWS. For example, a cohort study of Asian female breast cancer patients revealed that those receiving neoadjuvant chemotherapy had a 2.96-fold higher risk of developing AWS with shoulder mobility limitations (OR = 2.96; 95% CI = 1.25–6.98; P = 0.013) compared to untreated patients (29). Neoadjuvant chemotherapy may increase AWS risk through mechanisms such as immune function impairment and alterations in local tissue microenvironments. Chemotherapeutic agents could damage lymphatic endothelial cells, thereby compromising lymph drainage and promoting AWS onset. However, some studies failed to demonstrate a significant correlation between neoadjuvant chemotherapy and AWS occurrence. Such discrepancies may stem from variations in sample sizes or specific chemotherapy regimens, warranting further large-scale, high-quality studies to clarify this relationship.

The number of axillary lymph node dissections (ALND) and the number of positive lymph nodes may influence the development of AWS. A higher number of dissected lymph nodes reflects broader surgical damage to the axillary lymphatic system, increasing AWS likelihood. Zhang Linhui’s study demonstrated that patients with >15 dissected lymph nodes faced a 5.54-fold higher AWS risk compared to those with ≤3 nodes (30). Similar findings were reported by Fu Ting (14) and Wang Zhichun (31). Research indicates a rising trend in AWS incidence with increasing axillary lymph node dissection (ALND) extent, though the precise dose-response relationship remains unestablished (29). Additionally, the number of positive lymph nodes may correlate with AWS development. Positive nodes may trigger local inflammation and immune responses, inducing perilynphatic tissue fibrosis and elevating AWS risk. A retrospective analysis revealed that patients with tumor N ≥ 1 (presence of positive nodes) had significantly increased AWS risk (OR = 3.7, P < 0.001) (25). Nevertheless, mechanistic studies on how ALND extent and positive node numbers modulate AWS remain scarce, necessitating further investigation.

Body Mass Index (BMI) has been identified as a potential factor influencing the development of AWS, though the direction of association remains inconsistent across studies. In our cohort, patients with BMI < 24 kg/m² had a higher risk of AWS. This finding aligns with studies suggesting that lower BMI is associated with increased AWS risk (32, 33), while other reports have found higher BMI to be a risk factor (34).These contradictory findings may reflect two distinct phenomena that are difficult to disentangle. First, detection bias may contribute to the higher observed AWS risk in patients with lower BMI: these individuals have less subcutaneous adipose tissue, making subcutaneous cord-like structures more palpable and visible during physical examination. This mechanical advantage could lead to higher detection rates rather than truly higher biological risk. Second, true biological mechanisms may also play a role: patients with lower BMI have reduced buffering capacity against lymphatic vessel injury due to less protective adipose tissue, potentially leading to impaired postoperative compensatory mechanisms, increased risk of lymphatic fluid accumulation, and susceptibility to lymphatic reflux (32, 33). Conversely, higher BMI is associated with pro-inflammatory adipokine activity, which could promote systemic inflammation and fibrotic processes (34).The conflicting findings in the literature likely reflect a combination of these detection and biological factors, which may vary across studies depending on assessment methods, population characteristics, and surgical techniques (32–34). Future studies employing standardized assessment protocols and imaging techniques may help clarify the relative contributions of detection bias versus biological mechanisms in the relationship between BMI and AWS.

Surgical approach in breast cancer is closely associated with AWS development. Specifically, mastectomy (particularly radical mastectomy), due to its extensive surgical field and significant trauma to the axillary and surrounding tissues, may increase AWS risk. A retrospective study demonstrated that patients subjected to mastectomy had a significantly higher incidence of AWS compared to those undergoing other surgical approaches (p < 0.05) (25). Bergmann et al. conducted comprehensive physical examinations on 185 women 45 days post-breast cancer surgery and found that mastectomy patients had a twofold higher risk of AWS compared to those who underwent breast-conserving surgery (28). This may be attributed to the inevitable damage to axillary lymphatic vessels, blood vessels, and peripheral nerves during mastectomy, which disrupts lymphatic drainage and promotes tissue fibrosis, subsequently triggering AWS. In contrast, breast-conserving surgery combined with sentinel lymph node biopsy minimizes axillary trauma and may reduce AWS incidence. Nevertheless, the exact risk differences remain influenced by multiple factors, including surgical techniques and adjuvant therapies, necessitating further research to elucidate the specific association between surgical modalities and AWS.

Age plays a significant yet controversial role in AWS occurrence. Some studies suggest that younger patients are more susceptible to AWS (35), while others report higher risk in older patients (26). These conflicting findings may reflect a bimodal risk pattern: younger patients may mount a more vigorous inflammatory response to surgical trauma, leading to increased fibrous band formation during tissue healing. In contrast, older individuals may have age-related decline in physiological function, impaired lymphatic circulation, and reduced tissue repair capacity, which could paradoxically increase AWS risk through delayed recovery mechanisms. Younger patients, with their heightened metabolic activity and vigorous inflammatory responses to surgical trauma, may be more susceptible to fibrous band formation during tissue healing. In contrast, older patients may face increased AWS risk due to age-related decline in physiological function, impaired lymphatic circulation, and reduced tissue repair capacity.

Hypertension and diabetes as common chronic conditions may be associated with the occurrence of axillary web syndrome (AWS). Although research in this field remains limited, existing evidence suggests that these conditions may increase AWS risk by affecting vascular and neurological function. Previous studies have demonstrated that hypertension (HTN) correlates with an elevated risk of AWS, whereas diabetes mellitus (DM) may reduce this risk (12). The study by Sangahde et al. did not confirm an association between DM and AWS but reported a significant correlation between HTN and AWS: the risk of AWS in HTN patients was 2.72 times higher than in non-HTN individuals (8). Hypertension may impair local axillary blood circulation and lymphatic drainage by inducing vascular endothelial dysfunction, while diabetes could trigger neuropathy and microvascular complications, compromising tissue healing and increasing fibrotic propensity, thereby elevating AWS risk. However, current evidence regarding the relationship between hypertension, diabetes, and AWS remains insufficient, warranting targeted mechanistic studies to elucidate their association.

Radiotherapy, as a critical component of comprehensive breast cancer treatment, exhibits a controversial relationship with AWS. Some studies suggest that radiotherapy may increase AWS risk: a retrospective case-control study demonstrated a significantly higher incidence of AWS in breast cancer patients subjected to radiotherapy compared to the non-radiotherapy group (p < 0.05) (25), consistent with prior findings (18). However, subsequent research has indicated a protective effect of radiotherapy against AWS (36). Radiotherapy may contribute to AWS by damaging local axillary lymphatic vessels and blood vessels, leading to lymphatic reflux obstruction and tissue fibrosis. Additionally, it may induce localized inflammatory responses, exacerbating tissue injury and fibrosis. Nevertheless, other studies have not identified a significant association between radiotherapy and AWS, possibly due to variations in radiation dose, field, and timing. Differences in radiotherapy protocols and their distinct impacts on axillary tissues may account for these discrepancies (18, 25, 36). Radiotherapy may play a dual role: as an acute inflammatory trigger, it can damage local lymphatic vessels and blood vessels, leading to lymphatic reflux obstruction. As a promoter of chronic fibrosis, it may induce long-term tissue remodeling that could either exacerbate or, paradoxically, stabilize axillary structures. These opposing mechanisms may explain why some studies report increased risk while others suggest a protective effect.

Our study identified the tumor site (left-sided versus right-sided breast cancer) as a risk factor for postoperative AWS. These findings align with previous research; literature (30) reported that left-sided breast cancer patients had a 2.42-fold higher risk of AWS compared to right-sided patients. This laterality difference may influence postoperative complications through anatomical variations in lymphatic drainage or surgical technical disparities. Given the proximity of the left breast to the cardiac region, coupled with surgical procedural factors (e.g., potential technical variations during left axillary dissection by right-handed surgeons), lymphatic system injury or fibrosis severity may differ. However, whether this association stems from biological factors (e.g., lateralized immune response differences) or technical bias requires further mechanistic investigation. Multicenter cohort validation employing standardized surgical plans would strengthen this observation. If confirmed, tumor laterality could serve as a preoperative risk factor for AWS, facilitating targeted preventive measures.

The influence of adjuvant chemotherapy on the occurrence of AWS remains inconclusive. Adjuvant chemotherapy aims to eliminate residual tumor cells postoperatively and improve patient survival, yet chemotherapeutic agents may exert toxic effects on normal tissues. Some studies hypothesize that adjuvant chemotherapy could increase AWS risk by impairing axillary lymphatic circulation and tissue healing processes—chemotherapeutic agents may damage lymphatic endothelial cells, leading to lymphatic drainage dysfunction and promoting fibrotic formation. However, current research investigating the relationship between adjuvant chemotherapy and AWS is limited, with inconsistent conclusions. For instance, one study in breast cancer patients found no significant correlation between adjuvant chemotherapy and AWS incidence (p > 0.05) (26). These discrepancies may stem from variations in chemotherapy regimens and individual patient responses. Future large-scale, multicenter studies are warranted to clarify the precise impact of adjuvant chemotherapy on AWS. Of note, as both radiotherapy and adjuvant chemotherapy were initiated after surgery and may have occurred after AWS onset, their observed associations should be interpreted as exploratory rather than causal.

Among the nine predictive models systematically evaluated, the logistic regression (LR) model demonstrated the highest area under the curve (AUC = 0.72) and accuracy (0.76), with its interpretability and stability making it a reliable baseline model. The model is designed for use in the early postoperative period, when complications such as lymphedema and shoulder dysfunction often first become detectable. While the model’s low recall (0.28) limits its use as a standalone screening tool, it can serve as a decision-support tool to guide risk-stratified interventions: based on DCA, patients with predicted risk ≥20% may be prioritized for early physical therapy consultation within the first postoperative week, while those with lower risk can receive routine follow-up. Several factors may explain why LR, a relatively simple model, outperformed more complex algorithms. First, the modest sample size and event count (176 AWS cases) may limit the ability of complex ensemble methods to generalize without overfitting. Second, the predominance of categorical and binary risk factors may reduce the nonlinear advantages of tree-based models. Third, class imbalance (26.9% AWS prevalence) may disproportionately affect algorithms optimizing for overall accuracy. Fourth, while hyperparameter tuning was performed using grid search with 5-fold cross-validation, the sample size may have constrained its effectiveness for complex models. The low recall (0.28) reflects these challenges—the model would miss over 70% of AWS patients, a shortcoming that could delay essential interventions. Future iterations could explore resampling or cost-sensitive learning to improve sensitivity. These observations highlight that model complexity does not always translate to improved clinical utility, and that interpretable models like LR remain valuable where transparency and stability are prioritized.

The calibration and decision curve analyses provide important context for interpreting our model’s clinical value. The good calibration (Hosmer-Lemeshow p = 0.41) indicates that predicted risk probabilities align well with observed outcomes, which is essential for clinical decision-making where accurate risk estimation is needed. Furthermore, the positive net benefit across threshold probabilities from 10% to 45% suggests that the model could usefully guide interventions in clinical practice. For example, at a 20% threshold (where clinicians would intervene if predicted risk exceeds 20%), using the model would yield better outcomes than intervening on all patients or none, while avoiding unnecessary interventions in low-risk patients. This finding addresses concerns about the model’s clinical applicability and supports its potential role in postoperative risk stratification.

Several recent studies have used machine learning combined with SHAP to address different clinical questions in oncology. In breast cancer, this approach has been applied to predict neoadjuvant chemotherapy response and DCIS recurrence, as well as to assess osteoporosis risk in survivors (37–39). Similar methods have also been used in pancreatic and ovarian cancer research (40, 41). These studies show that SHAP can help make machine learning models more interpretable by identifying which factors contribute most to the predictions. Our study adopts the same approach to identify factors associated with AWS in postoperative breast cancer patients, providing clinicians with a clearer understanding of relevant risk factors.

## Limitations

This study has several limitations. First, its retrospective nature and single-center design may limit the representativeness of the sample and introduce potential selection bias and risks associated with incomplete medical records. Second, the diagnosis of AWS was based on clinical physical examination. Although standardized procedures referenced from previous studies were followed, the assessment could still be subject to the evaluator’s subjectivity. Third, the prediction model incorporated postoperative complications (e.g., lymphedema, shoulder dysfunction), which imposes a temporal logical constraint for preoperative early warning. The model is therefore intended for postoperative risk stratification rather than preoperative prediction. In the early postoperative period, when these complications often first become detectable, the model can help identify patients who may benefit from intensified surveillance or early intervention. Future research could explore models based solely on preoperative and intraoperative features for earlier risk stratification. Additionally, adjuvant chemotherapy and radiotherapy were included as exploratory variables but may have occurred after AWS onset in some patients; their associations should therefore be interpreted with caution. Fourth, the model was only validated internally and has not undergone external independent validation; therefore, its generalizability remains unclear. While we have now included calibration and decision curve analyses to assess model fit and clinical utility, these analyses were performed on the same test set and require validation in independent cohorts. Additionally, the relatively small sample size in certain subgroups (e.g., advanced clinical stage) precluded robust subgroup analyses; including subgroup analyses stratified by clinical stage and surgical type; future studies with larger, multicenter cohorts should evaluate model performance across diverse patient populations to better support precision medicine. Future research should focus on external validation and prospective evaluation of the model’s impact on clinical outcomes. Finally, although the logistic regression model demonstrated good performance in terms of AUC and accuracy, its recall rate was relatively low, which may lead to high-risk patients being underdiagnosed. In clinical application, it should be used in conjunction with other assessment tools for comprehensive judgment.

## Conclusion

This study identified 14 highly predictive features for AWS using LASSO regression. SHAP value analysis revealed that the type of lymph node surgery and lymphedema status were the most profound predictive variables. After partitioning the dataset into a development set and test set at a 7:3 ratio, comparative analysis of nine algorithms demonstrated that the LR model achieved optimal AWS prediction performance (accuracy = 0.76, AUC = 0.72). The LR model demonstrated acceptable discrimination (AUC = 0.72) for postoperative AWS risk identification. However, its low recall indicates that it is not suitable as a standalone screening tool. Instead, the model may serve as a decision-support tool to complement clinical evaluation, helping clinicians identify patients who may benefit from closer monitoring and early intervention. To address the limitation of single-center data, our next steps include external validation in independent multicenter cohorts and prospective evaluation of the model’s clinical utility in guiding postoperative care.

## Data Availability

The original contributions presented in the study are included in the article/**Supplementary Material**. Further inquiries can be directed to the corresponding author.
